# Preoperative short-course radiation therapy with PROtons compared to photons in high-risk RECTal cancer (PRORECT): Initial dosimetric experience

**DOI:** 10.1016/j.ctro.2022.100562

**Published:** 2022-12-17

**Authors:** Cristiana Pedone, Bruno Sorcini, Caroline Staff, Johanna Färlin, Tone Fokstuen, Jan-Erik Frödin, Per J. Nilsson, Anna Martling, Alexander Valdman

**Affiliations:** a Department of Radiation Oncology, Karolinska University Hospital, Stockholm, Sweden; b Department of Oncology and Hemato-oncology, University of Milan, Milan, Italy; c Department of Radiotherapy Physics and Engineering, Medical Physics and Nuclear Medicine, Karolinska University Hospital, Stockholm, Sweden; d Department of Oncology, Capio S:t Göran Hospital, Stockholm, Sweden; e The Skandion Clinic, Swedish National Proton Beam Therapy Facility, Uppsala, Sweden; f Division of Oncology, Department of Pelvic Cancer, Karolinska University Hospital, Sweden; g Upper GI Unit, Theme Cancer, Karolinska University Hospital, Stockholm, Sweden; h Division of Coloproctology, Department of Pelvic Cancer, Karolinska University Hospital, Sweden; i Department of Molecular Medicine and Surgery, Karolinska Institutet, Stockholm, Sweden; j Department of Oncology and Pathology, Karolinska Institutet, Stockholm, Sweden

**Keywords:** Proton, Rectal cancer, Short-course, Toxicity, Dosimetric comparison

## Abstract

**Background and purpose:**

Neoadjuvant short-course radiotherapy (SCRT) followed by full-dose systemic chemotherapy is an established treatment modality in locally advanced rectal cancer (LARC). Until recently, SCRT has been exclusively delivered with photons. Proton beam therapy (PBT) may minimize acute toxicity, which in turn likely impacts favorably on the tolerability to subsequent chemotherapy. The aim of this study is a dosimetric comparison between SCRT with photons and protons in the randomized phase II trial PRORECT (NCT04525989).

**Materials and methods:**

From June 2021 to June 2022, twenty consecutive patients with LARC have been treated according to study protocol. For each patient, both a VMAT and a PBT treatment plans have been generated and compared pairwise.

**Results:**

Dose-volume histogram (DVH) analysis revealed that SCRT with protons significantly reduced radiation dose to pelvic organs at risk including bladder, bones, and bowel in comparison to SCRT with photons. Photon and proton treatment plans had equivalent conformity and homogeneity indexes.

**Conclusion:**

Preoperative SCRT with protons offers a significant reduction of radiation dose to normal tissues compared with current photon-based radiotherapy technique. Demonstrated dosimetric advantages may translate into measurable clinical benefits in patients with LARC. Clinical implications of the dosimetric superiority of SCRT with protons will be presented in the coming reports from the PRORECT trial.

## Highlights

• The first randomized trial comparing short-course radiotherapy with photons or protons in primary treatment of locally advanced rectal cancer.
• Twenty patients successfully treated during the first year.
• Excellent dosimetric quality of proton treatment plans.
• Significantly less irradiation of pelvic OAR with proton beam therapy.

## Graphical abstract

**Figure:**
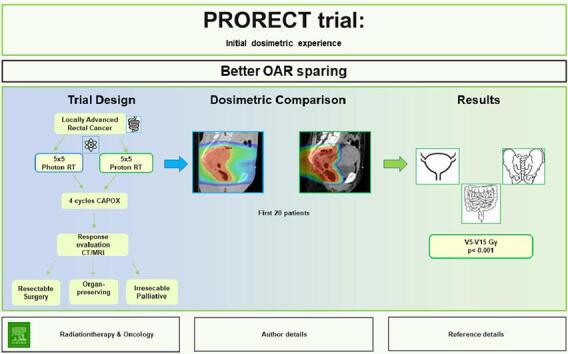

## Introduction

The standard of care for locally advanced rectal cancer (LARC) has included preoperative chemoradiation, total mesorectal excision (TME) surgery and selective post-operative adjuvant chemotherapy [1]. Total neoadjuvant therapy (TNT) is a novel approach for LARC, which delivers both systemic chemotherapy and neoadjuvant (chemo)radiotherapy prior to surgery [2]. There is a growing interest in treating LARC with TNT after recently published randomized phase III trials RAPIDO [3], PRODIGE-23 [4] and STELLAR [5].

SCRT and long-course chemoradiation therapy (LC-CRT) are the two established preoperative radiotherapeutic modalities that are equally effective in lowering risk of developing local recurrencies [6], [7], [8], [9], [10]. SCRT for rectal cancer was pioneered in Sweden [11] and has since gained increased acceptance in treatment of LARC. Recent ESMO [12] and NCCN [13] guidelines list either SCRT or LC-CRT followed by systemic chemotherapy as options for preoperative treatment in LARC.

Despite great improvements over the past decades, even the most technically advanced radiotherapy delivers a significant amount of radiation to organs at risk (OAR) outside treatment target, resulting in acute and late radiation-induced toxicity [14], [15]. OAR for patients with rectal cancer include bladder, bowel, pelvic bone marrow, nervous tissue, skin, and genitalia. Acquired radiation dose to these OAR can result in adverse events which can affect survival and quality of life [16], [17], [18], [19]. As demonstrated in the RAPIDO trial, considerable toxicity was observed in the preoperative period with 48 % of patients in the SCRT arm experiencing grade 3+ toxicity [20]. Diminishing radiotherapy-induced toxicity could be the way to increase compliance of subsequent systemic chemotherapy, and favorably impact on disease-free survival (DFS) and metastatic disease. However, clinical evidence of these effects is still lacking.

Neoadjuvant radiotherapy in rectal cancer is currently photon-based. Proton beam therapy (PBT) has emerged as a potentially attractive treatment option for rectal cancer that may minimize dose to normal tissues and reduce treatment-related toxicity. The depth-dose characteristics of PBT allows for a steep fall-off of radiation dose just distal to the tumor target [21]. Comparative PBT treatment planning studies have shown effective sparing of the normal tissue in the pelvic area including bone marrow, bowel, and bladder in favor of proton therapy [22], [23], [24]. The role of PBT in neoadjuvant therapy for LARC is currently being studied in the Swedish National PRORECT trial (NCT04525989), the first ongoing randomized clinical trial for primary rectal cancer and PBT. In this early report we present the initial single-institution treatment planning results of PRORECT trial.

## Materials and methods

### Study design

**Fig. 1:**
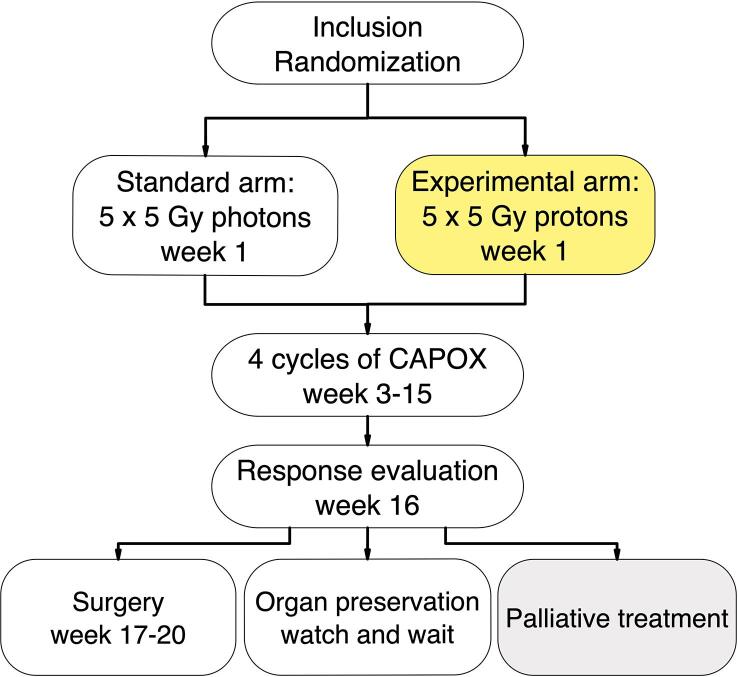
PRORECT study treatment algorithm.

PRORECT is a Swedish national two-arm prospective randomized multicenter phase II trial. (Fig. 1). The study compares preoperative SCRT with photons or protons for treatment of LARC. The primary outcome measure is acute grade 2+ gastrointestinal toxicity measured before planned start of the third (3) chemotherapy cycle. Tolerability of preoperative chemotherapy and overall toxicity are included among the secondary endpoints.

Protocol details can be found in supplementary Protocol Synopsis, Radiotherapy Appendix, Target volumes Appendix, Radiology Appendix and prorect.se.

Patients with LARC with high risk of systemic recurrence defined by the presence of at least one high-risk feature on pre-therapeutic magnetic resonance imaging (MRI) (T4 stage; N2 stage; positive mesorectal fascia; extramural vascular invasion; positive lateral lymph node) [25] are randomized (1:1) to receive SCRT to a total dose of 25 Gy relative biologic effectiveness (RBE)*, (*applies to all doses in Gy later in text) in five daily fractions with either photons or protons, followed by a standard systemic chemotherapy consisting of minimum four cycles of Capecitabine and Oxaliplatin (CAPOX).

### Ethics

PRORECT has received approval from the Swedish Ethical Review Authority (Dnr 2020–02192). Written informed consent, signed, and dated, was obtained from each patient before inclusion in the trial.

### Radiotherapy

Karolinska University Hospital as the first participating center followed internal benchmarking as well as quality assurance (QA) procedures (Radiotherapy Appendix). To minimize within-center variability, all target volume and OAR delineations have been performed by a single radiation oncologist (AV). The photon and proton plans have been generated by the site’s principal medical physicist (BS), centrally reviewed, and approved at the national proton therapy boards.

#### Definition of target volumes and dose constraints

All patients performed both CT and MRI planning scans in supine position combined with standardized bladder-filling protocol. MRI-based target delineation was on T2-weighted imaging. The CTV included the GTV and all involved lymph nodes, presacral nodes, the complete meso-rectal fascia and internal iliac lymph nodes. In certain cases, external and inguinal iliac nodes, as well as fossa ischiorectalis were included in accordance with the European guidelines [26] (Target volumes Appendix). For photon treatment plans, PTV was generated by adding a 3D isotropic margin of 6 mm to the CTV. The dose to the OAR was aimed to be as low as reasonably achievable (ALARA) and comply with the following constraints: bowel bag V18Gy ≤ 450 cc; femoral heads Vmean <25 Gy, sacrum (spinal canal at the level of S1-S2) V25%<60 %, bladder and pelvic bones ALARA, avoiding hotspots (Radiotherapy Appendix).

#### Characteristics of planning procedure and treatment technique

Both photon and proton treatment plans were generated, optimized, and analyzed using Eclipse Treatment Planning System (version 16.01.10, Varian Medical Systems, Palo Alto, CA, USA) according to current ICRU recommendations [27]. Reference dosimetry was carried out according to the IAEA report TRS 398 [28]. The absorbed dose in the patient geometry was calculated by using validated algorithms: for the photon plan with the anisotropic analytical algorithm (AAA version 16.1.0) and for the proton plans with the algorithm PCS (version 15.6.04). The dose grid voxel dimensions were 2.5x2.5x2 mm^3^.

Photon treatments were given with TrueBeam ® linear accelerators (Varian Medical Systems) using RapidArc ® technique with two arcs, and a photon energy of 6 MV. Proton treatments were delivered with an Ion Beam Applications (IBA Proteus®PLUS) powered facility, exclusively using the pencil beam scanning technique.

PBT robustness optimization and evaluation was performed for 14 scenarios. The first 12 scenarios used 6 mm isocenter displacements along the cardinal axes with a ± 3.5 % density change. The last 2 scenarios only took a ± 3.5 % density change into account. CTV dose coverage fulfilled robustness evaluation scenarios (Radiotherapy Appendix).

One isocenter was used and a coplanar beam arrangement was adopted using two posterior oblique beams (150°±5° and 210°±5°). Spot spacing was set to 3 mm. Most plans were optimized using the single field uniform dose (SFUD) technique. The choice of multi-field optimization (MFO) technique was mainly governed by irradiation of inguinal lymph nodes. In four cases, comparative 3-field treatment plans were generated (Fig. 4). However, all PBT treatments were delivered with 2-field technique and no plan made use of a range shifter. The RBE value of 1.1 was used for protons and the prescribed dose is the corresponding dose for photons in Gy (RBE) [29]. Rectal gas was contoured, and the dose was computed with and without a Houndsfield unit (HU) override to 0, which corresponds to water equivalent tissue.

In both treatment arms, IGRT was employed by verifying the position of the patient on daily CBCT. Additionally, the optical surface scanning system Catalyst ™ (C-RAD, Uppsala, Sweden) was used as a complement for positioning and intrafractional monitoring of the patient (Radiotherapy Appendix). Treatment fractions in both study arms have been given on five consecutive weekdays starting on Mondays.

#### Evaluation of dosimetric parameters

For each patient, both photon and proton treatment plans were generated. To facilitate comparison between the photon and proton target coverage, we compared CTV coverage and dose to OARs pairwise and analyzed at maximum dose (Dmax), minimum dose (Dmin) and mean dose (Dmean) values. Additionally, homogeneity index (HI) [30] and conformity index (CI) [31] were evaluated for CTV. The HI was expressed in terms of the standard deviation and of D2–D98% according to ICRU Report 83 [32]. The conformality of the plans was measured with a CI, with CI95% defined as the ratio of the target volume covered by the 95 % isodose line divided by the total volume.

The OAR analyses included: Volume, Dmax, Dmin and Dmean. Furthermore, the absolute OAR volume receiving 5 Gy, 10 Gy, 15 Gy and 25 Gy were assessed for bladder, bones, and bowel bag.

### Statistical analysis

In each patient, dosimetric results for two irradiation techniques were compared using paired, two-tailed Wilcoxon Signed-Rank test. Results were considered statistically significant with p < 0.05.

## Results

From June 2021 to June 2022, twenty consecutive patients with LARC have been treated in the PRORECT trial. Ten patients in standard arm received routine VMAT photon treatment at the Karolinska University Hospital in Stockholm, Sweden. Ten patients in experimental arm received SCRT with PBT at the Swedish National proton facility Skandionkliniken in Uppsala. None of the original treatment plans have been re-planned.

The median age of the patients was 57 years. Baseline clinical staging is summarized in Table 1.

**Table 1:** Baseline clinical characteristics of the patients included in PRORECT trial.

Characteristic, number (%)	Protons	Photons
Number of patients	10 (50)	10 (50)
Age (median, range)	59 (40–67)	54 (36–73)

Gender		
Female	4 (40)	4 (40)
Male	6 (60)	6 (60)

T-stage		
T2	1 (10)	–
T3	5 (50)	4 (40)
T4	4 (40)	6 (60)

N-stage		
N1	4 (40)	2 (20)
N2	6 (60)	8 (80)

MRF-status		
MRF+	5 (50)	5 (50)
MRF-	5 (50)	5 (50)

EMVI-status		
EMVI+	6 (60)	7 (70)
EMVI-	4 (40)	3 (30)
		
Lateral LN+	0 (0)	0 (0)

The mean CTV volume was 812 cm^3^. Dosimetric parameters of GTV and CTV for photons and protons are presented in Table 2. The dosimetric evaluation of CI and HI for CTV showed that the two irradiation techniques are equivalent (CI 0.99 and 1.00; HI 0.04 and 0.03 for photons and protons, respectively). Comparative dosimetric results for pelvic OARs are presented in Table 3.

**Table 2:** Dosimetric parameters of GTV and CTV for VMAT (X) and PBT (P).

Vmean (cm^3^) ± SD	GTV (50.4 ± 35.2)		CTV (811.5 ± 233.8)	
Variable (mean ± SD)	X	P	X	P
Dmin (%)	99.7 ± 0.7	97.4 ± 0.8	96.4 ± 1.1	97.2 ± 1.0
Dmax (%)	102.4 ± 1.1	102.6 ± 0.6	104.5 ± 0.9	103.0 ± 0.9
Dmean (%)	101.1 ± 0.7	99.9 ± 0.5	100.4 ± 0.4	100.2 ± 0.4
Conformity index			0.99 ± 0.05	1.00 ± 0.00
Homogeneity index			0.04 ± 0.01	0.03 ± 0.01

**Table 3:** Comparative dosimetric results for pelvic OARs. VMAT (X) vs PBT (P+).

Vmean (cm^3^ ± SD)	V5 Gy			V10 Gy			V15 Gy			V25 Gy		
	X	P+	p	X	P+	p	X	P+	p	X	P+	p
Bladder (274.7 ± 162.0)	268.4 ± 152.1	127.5 ± 75.4	*<0.001*	185.6 ± 102.2	99.0 ± 63.2	*<0.001*	109.8 ± 62.1	78.6 ± 53.1	*<0.001*	12.0 ± 12.2	6.6 ± 13.7	0.22
Pelvic bones (1660 ± 314.5)	809.4 ± 204.7	420.9 ± 160.3	*<0.001*	593.8 ± 166.2	338.1 ± 125.0	*<0.001*	333.2 ± 93.6	228.4 ± 66.0	*<0.001*	30.6 ± 12.8	38.4 ± 15.9	0.09
Bowel bag (964.4 ± 403.2)	712.8 ± 329.7	247.1 ± 132.3	*<0.001*	461.5 ± 230.9	182.9 ± 102.9	*<0.001*	215.3 ± 136.4	141.2 ± 83.6	*<0.001*	17.9 ± 18.2	23.6 ± 26.3	*<0.05*

**Fig. 2:**
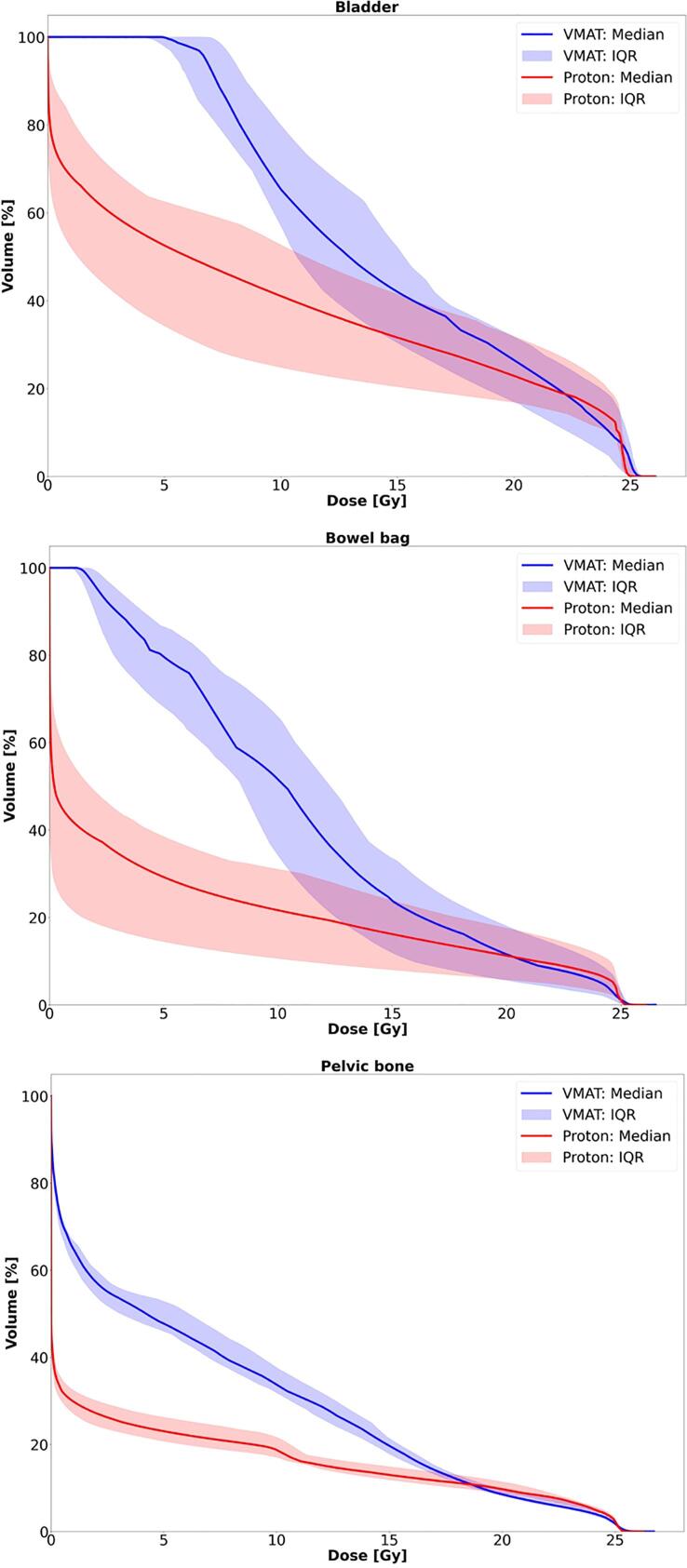
DVHs for bladder, bowel bag and pelvic bone. Solid curves are the median values for all patients, and shaded region indicate the 25th to 75th percentiles (IQR). Photon VMAT (blue), PBT (red).

**Fig. 3:**
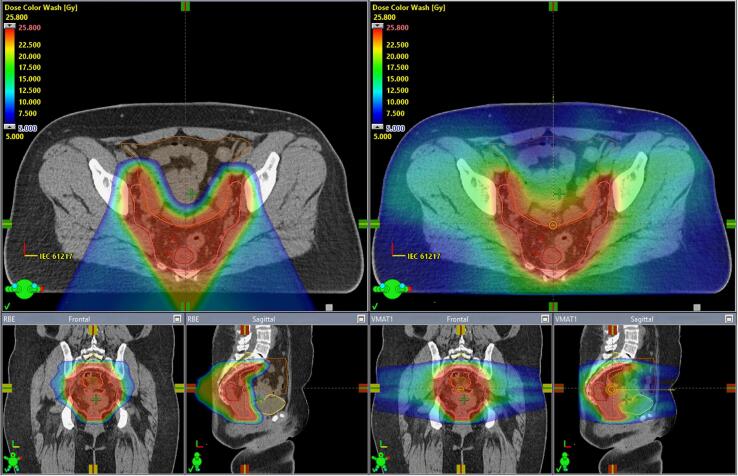
Color wash dose distribution of PBT (left) and VMAT (right) treatment plans. The color wash interval is set to 5–25.8 Gy of the prescribed dose. CTV, GTV, bladder, bowel bag and spinal canal at the level of S1-S2 are outlined as a solid pink, red, dark yellow, brown and light-yellow lines, respectively.

**Fig. 4:**
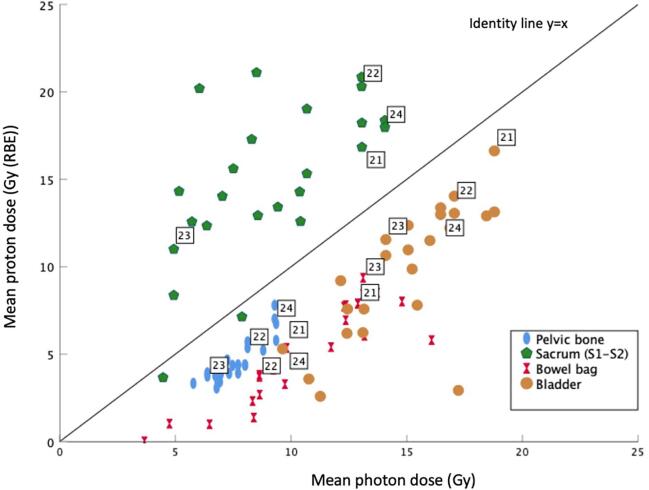
Scatter plot for mean photon dose (Gy) versus mean proton dose (Gy (RBE)). Pelvic bone (blue), S1-S2 (green), bowel bag (red) bladder (yellow). For each OAR, cases 21–24 represent values for 3-field technique.

Dose-volume histograms (DVH) for OARs are shown in Fig. 2. Comparative treatment plans are shown in Fig. 3. Scatter plot (mean DVHs for photons and protons for three OARs) is presented in Fig. 4. In all four cases, the values for comparative 3-field plans were on the same side of the identity line.

### OAR specific dosimetric results

#### Bladder

The mean bladder volume was 274.7 cm^3^. The proton plans achieved a significant reduction of irradiated bladder volume at 5, 10 and 15 Gy levels (Wilcoxon S-R test, Z: −3.9 for 5 and 10 Gy and Z: −3.6 for 15 Gy, p < 0.001), (Table 3) and significantly lower values at Dmin and Dmean (Supplementary table).

#### Bone

The mean volume of the irradiated bone was 1660.8 cm^3^. Proton plans resulted in systematic reduction of irradiated bone volume over the entire dose range. Significant advantage for protons was detected at 5, 10 and 15 Gy values (Table 3) as well as Dmin and Dmean (Supplementary table).

#### Bowel bag

The mean volume of the bowel bag was 964.4 cm^3^. Significant sparing was achieved for all measured values (Wilcoxon S-R test, Z: −3.9 for 5 and 10 Gy, −3.8 for 15 Gy, p < 0.001 and Z: −2.1 for 25 Gy, p < 0.005) (Table 3). Significant advantage for protons was observed at Dmax, Dmin and Dmean (Supplementary table).

#### Femoral heads

The proton plans spared femoral heads at Dmax, Dmin and Dmean (Supplementary table).

#### Sacrum (spinal canal at the level of S1-S2)

The Dmax and Dmean values were higher for proton plans: photons vs protons: Dmin 3.8 vs 2.7 Gy, Dmax 17.6 vs 23.2 Gy, Dmean 8.6 vs 14.6 Gy (Supplementary table).

## Discussion

Prospective randomized clinical trials comparing radiotherapy with photons and protons in the treatment of rectal cancer have long been awaited. To the best of our knowledge, PRORECT (NCT04525989) is the first randomized phase II trial comparing radiotherapy delivered with protons or photons as part of the neoadjuvant treatment for LARC. PRORECT is ongoing and currently recruiting from three centers in Sweden. As of now, twenty patients have been randomized and treated during the first year. In this early report based on the initial 20 patients we report significant dosimetric advantages with PBT compared to photons.

There are several studies demonstrating dosimetric advantages for protons in the pelvic area [22], [33], [34], [35], [36]. A recent metanalysis [37] showed improved dosimetric radiation profile with PBT compared to intensity modulated radiation therapy (IMRT) in treatment of rectal cancer. However, no comparative planning studies have previously been done as part of the ongoing randomized PBT clinical trial in rectal cancer*.*

Radiotherapy with protons in this study offered excellent target coverage and plan robustness that was equivalent to RapidArc® treatment plans. At the same time, PBT resulted in significantly reduced irradiation of healthy tissue. Significant sparing of the bladder, bones and bowel could be achieved in majority of the measured DVH values with the most pronounced difference in the lower and middle dose range.

Following the results from the RAPIDO trial, SCRT and subsequent chemotherapy has emerged as a valid option for patients with LARC. Clinical implications of the radiation-induced toxicity in this setting are twofold: acute toxicity per se may lead to the abortion of the treatment [38], [39]. As demonstrated in Stockholm III study, radiation-induced toxicity related to SCRT alone required preoperative hospitalization in 7 % of the patients [39]. Secondly, acute radiation-induced toxicity may negatively impact on compliance to chemotherapy. Gastro-intestinal toxicity is the most common adverse effect in the neoadjuvant period [20] and is a combination of radiation-induced and chemotherapy-related toxicity. Taking into account that the volume of bowel exposed to radiation is predictive of toxicity even at low doses [40], limiting radiation exposure of the bowel appears reasonable.

Pelvic bone is the primary site of hematopoiesis in adults [41]. As demonstrated here, PBT effectively reduces doses to pelvic bone which may lead to decreased hematologic toxicity. An association between myelosuppression and bone volume irradiated with doses ranging from 5 to 40 Gy has been recognized [42], [43], [44]. Thus, less hematologic toxicity with proton therapy may positively impact on tolerability of following chemotherapy.

Moreover, as showed by the Danish Rectal Cancer Group, preoperative radiotherapy interferes with several aspects of urinary and sexual functioning [45]. As many as 63 % of patients reported symptoms of urgency and incontinence following rectal surgery which was significantly exacerbated by radiotherapy. Significant sparing of the bladder demonstrated here can therefore improve genitourinary adverse effect profile.

It has to be noted that PBT in this study resulted in higher doses to spinal canal at the level of S1-S2, as compared to photon treatments owning to the fact that PBT planning was done using two posterior oblique beams. However, these higher values were well within dose constraints. No adverse effects related to sacral plexus or pelvic insufficiency fractures have yet been reported in the proton arm of the PRORECT trial (unpublished data). Given the absence of validated dose constraints to pelvic bone [46], we do not expect these results to have clinical relevance.

Clinical use of proton therapy has the potential to minimize the induction of secondary malignancies due to modified low-dose areas and steep dose gradients [47]. A recent Dutch study demonstrated that patients who received RT for a previous pelvic cancer were at increased risk for rectal cancer [48]. A meta-analysis with focus on radiotherapy for prostate cancer showed significantly increased risk of therapy-related rectal cancer [49]. Even though no increased risk of second primary cancer following standard preoperative radiotherapy for rectal cancer has been demonstrated in an epidemiological study [50], the possibility of these effects may still exist after irradiation with higher doses and/or longer follow-up, given the increasing incidence of rectal cancer among younger generations [51], significant increase in younger patients presenting with LARC and an increasing number of rectal cancer survivors [52].

Finally, recent advantages in neoadjuvant primary treatment of LARC can potentially lead to treatment de-escalation. Since radiation dose-escalation can improve tumor regression [53], more patients who achieve a complete clinical response can be considered for ‘watch and wait’ surveillance, and therefore avoid rectal cancer surgery [54], [55]. However, dose-escalation comes at a price of higher radiation-induced toxicity [53]. In this setting, PBT has the potential to deliver higher radiation doses with less toxicity leading to better clinical response rates.

## Conclusions

For the first time, we present the results of the treatment planning study as part of the ongoing randomized proton trial PRORECT (NCT04525989). Our results show that PBT treatment plans achieved significantly less irradiation of OAR for rectal cancer compared to state-of-the-art photon-based radiotherapy. These solid dosimetric results further support the benefits of proton beam radiotherapy in the neoadjuvant treatment of LARC. The prospective randomized design of the PRORECT trial will allow to determine whether demonstrated dosimetric superiority of proton beam treatments can be translated into measurable clinical benefits for the patients with LARC.

## Declaration of Competing Interest

The authors declare that they have no known competing financial interests or personal relationships that could have appeared to influence the work reported in this paper.
