# Supplementary material for: Preoperative short-course radiation therapy with PROtons compared to photons in high-risk RECTal cancer (PRORECT): Initial dosimetric experience
Source: Clin Transl Radiat Oncol. 2022 Dec 17;39:100562. doi: 10.1016/j.ctro.2022.100562 (PMC9792362; doi:10.1016/j.ctro.2022.100562)
Supplement: Supplementary data 1 [file mmc1.docx]

**Supplementary table.** Dosimetric results for all analyzed OARs. Dose comparison between photons (X) and protons (P)

| Vmean (cm^3^) ± SD | Dmin (Gy, RBE) | | | Dmax (Gy, RBE) | | | Dmean (Gy, RBE) | | |
| --- | --- | --- | --- | --- | --- | --- | --- | --- | --- |
|  | X | P | p-value | X | P | p-value | X | P | p-value |
| Bladder  (274.7  ±162.0) | 5.5±  2.4 | 0.0±  0.1 | *<0.001* | 25.5±  0.4 | 25.3±  0.3 | 0.07 | 14.5±  2.5 | 9.0±  3.6 | *<0.001* |
| Body  (39073.7  ±11230.8) | 0 | | | 26.4±  0.2 | 26.0±  0.3 | *<0.005* | 4.2±  1.0 | 2.2±  0.9 | *<0.001* |
| Pelvic bones  (1660.8  ±314.5) | 0 | | | 26.1±  0.2 | 25.9±  0.2 | *<0.001* | 7.5±  0.9 | 4.4±  1.1 | *<0.001* |
| Bowel bag  (964.4  ±403.2) | 1.2±  0.6 | 0 | *<0.001* | 25.4±  2.5 | 24.6±  4.7 | *<0.005* | 10.5±  3.2 | 4.9±  2.7 | *<0.001* |
| Femoral head L  (51.6  ±13.8 ) | 5.2±  1.9 | 0.7±  2.3 | *<0.001* | 16.7±  2.8 | 14.6±  5.2 | *<0.05* | 9.4±  2.0 | 2.5±  4.3 | *<0.001* |
| Femoral head R  (50.3  ±14.1) | 5.0±  1.9 | 0.9±  2.7 | *<0.001* | 16.6±  2.5 | 12.9±  5.3 | *<0.005* | 9.4±  2.1 | 2.5±  4.2 | *<0.001* |
| Sacrum (S1-S2)  (14.8  ±4.2) | 3.8±  1.8 | 2.7±  4.8 | 0.08 | 17.6±  4.6 | 23.2±  1.7 | *<0.001* | 8.6±  2.8 | 14.6±  4.5 | *<0.001* |
