# Supplementary material for: Preoperative short-course radiation therapy with PROtons compared to photons in high-risk RECTal cancer (PRORECT): Initial dosimetric experience
Source: Clin Transl Radiat Oncol. 2022 Dec 17;39:100562. doi: 10.1016/j.ctro.2022.100562 (PMC9792362; doi:10.1016/j.ctro.2022.100562)
Supplement: Supplementary data 2 [file mmc2.pdf]

Radiology Appendix /Lennart Blomqvist

MRI

Technical Requirements

1.5T or 3T MR equipment

Phased-array receiver coils for pelvic-/body imaging

Preparations: Four hours fasting, rectal emptying with

Microlax or likewise prior to examinations

Antispasmodic agents advocated if no contraindications

No endoluminal or intravenous contrast agents

Image sequences

T2-weighted high-resolution sequences in at least three different planes (sagittal, transaxial and oblique planes) where at least one imaging sequence is perpendicular to the rectum at the level of the tumour interleaved without interslice gap with maximum 3 mm section thickness with a voxel size of maximum 3x0,8x0,6 mm (1). If low tumors, additional oblique sequences including the tumour parallel and perpendicular to the anal canal are performed with same prerequisites for spatial resolution.

An echo-planar diffusion weighted sequence of the pelvis should be performed in the same anatomical position as the axial T2-weighted sequence of the pelvis. Voxel size should not exceed 5x2x1,5 mm. The diffusion weighted sequence should include at least three b-values including the values b=0, 50 and 800 s/mm^2 as well as an ADC-map calculated from the b=50 and b=800 sequences.

T1-weighted axial sequence of the pelvis is suggested but not mandatory.

MRI reporting

Baseline examination before neoadjuvant treatment

Level of the tumour

The distance of the tumour from the anorectal junction and/or from the anal verge is measured by electronic calipers on sagittal images. The length of the tumour is measured and reported. It is also stated whether the tumour is above, at or below the level of the peritoneal reflection. For low tumours it is stated whether the tumour is within a mm from the levator muscles or not, whether there is involvement of the intersphincteric plane and the external sphincter.

Morphology

Morphology of the tumour is described whether the tumour is polypoid, (semi)annular. If there is evidence of a mucinous tumour indicated by typical high signal intensity on T2-weighted images, this is also reported.

Depth of extramural spread

The maximum depth of extramural depth from the outer edge of the muscle layer to the outer edge of the tumour is measured on high resolution images perpendicular to the rectum at the level of the tumour (1)

Extramural vascular invasion (EMVI+ if present)

EMVI is recorded when there is tumour extension along a vessel as a serpentiguous extension of tumour signal within a vascular structure. Criteria according to Smith et al are used to define whether extramural vascular invasion is present or not (2) .

Mesorectal Fascia

Potential involvement of the mesorectal margin is defined as tumour extending within 1 mm of the mesorectal fascia or closer. In low tumours, the mesorectal margin consists of the fascia covering the levator muscles.

Perforation of the peritoneal reflection by tumour

Peritoneal involvement is reported when nodular extension of tumour beyond the peritoneal reflection is found (3)

Mesorectal lymph node metastases

The total number of mesorectal lymph nodes is reported and the number of lymph nodes regarded as metastatic according to ESGAR consensus guidelines (12). In these quidelines, the size of the lymph nodes deteremines the number of morphological criteria needed (less than 5 mm three criteria, 5-8 mm two criteria and 9 mm or more with either irregular outer border or heterogeneous signal). To note is that a modification from these criteria is that at

least one malignant morphological criterium is required for a metastases regardless of lymph node size.

Extramesorectal lymph node metastases

Presence of suspected metastatic inguinal, lateral pelvic lymph nodes should be reported. Metastastic extramesorectal lymph nodes or pelvic sidewall lymph nodes are defined by morphological criteria same as mesorectal lymph nodes although it is important to note the presence, size and location of all extramesorectal lymph nodess.

Evaluation post chemo-irradiation

When MRI is performed after neoadjuvant treatment, the post treatment MRI is compared with MRI at baseline. Viable tumour (high signal intensity) is separated from post treatment fibrosis (low signal intensity) on T2-weighted images and/or presence of reamaing impeded diffusion on high b-value diffusion weighted images. For mucinous tumours, remaining or increasing pure mucin pools may not necessary indicates progressive disease.

Length of tumour and tumour and fibrosis if these are not clearly separated is measured as in baseline on sagittal T2-weighted images.

MR tumour regression grading based on T2-weighted images (mrTRG) is performed according to Patel et al (9). This is a five graded scale for this study modified to include diffusion weighted images as follows:

mrTRG1 - no remaining tumour on T2- or diffusion-weighted images (DWI) and normal bowel wall. **A remaining more or less flat fibrotic scar with homogeneous low T2-w signal intensity is common in these cases**

MrTRG2 -the majority of the tumour converted to fibrosis with homogeneous low signal intensity on T2-weighted images **with minor areas of impeded diffusion on high b-value DWI.**

MrTRG3 -mixture of more or less equal amounts of fibrosis and tumour signal with or without areas of restricted diffusion on high b-value DWI

mrTRG4 -corresponds to predominantly tumour signal with remaining restricted diffusion on high b-value DWI.

mrTRG5 - no visible treatment effect neither on T2-weighted images or or high b-value DWI

A clinical complete response (CR) or near CR is defined as mrTRG1-2 together with no or minor areas of impeded diffusion on high b-value diffusion weighted images and a clear reduction in tumour size post-pretreatment (10). To note is that remaining TRG2 after a second or third follow up MRI more than 8 weeks after completion of CRT should be regarded suspicious for non-complete response.

Regarding lymph nodes, the short axis diameter of mesorectal and extramesorectal lymph nodes is measured. Lymph nodes with malignant morphogical features pretreatment and a short axis diameter post treatment of equal to or more than 5 mm are considered malignant.

Note:

Role of volume reduction

The changes in size of tumour is only visually qualitatively assessed as the difference in length pre -post treatment and evaluated together with changes in signal on T2-weighted images and DWI.

(The length (L), width (W) and thickness(T) of the tumour can be measured and tumour volume (V) before (pre) and after (post) radiochemotherapy can be estimated as L x W x T /2. Percentage volume reduction of tumour after treatment can be calculated as 100x (Vpre-Vpost)/Vpre. A volume reduction of at least 80 % is highly predictive of CR but should be evaluated together with the other MR imaging findings (11).)

References:

1. Taylor FG, Swift RI, Blomqvist L, Brown G. A systematic approach to the interpretation of preoperative staging MRI for rectal cancer. AJR Am J Roentgenol. 2008 Dec;191(6):1827-35. Review.

2. Smith NJ, Shihab O, Arnaout A, Swift RI, Brown G. MRI for detection of extramural vascular invasion in rectal cancer. AJR Am J Roentgenol. 2008 Nov;191(5):1517-22. Review.

3. Brown G, Radcliffe AG, Newcombe RG, Dallimore NS, Bourne MW, Williams GT. Preoperative assessment of prognostic factors in rectal cancer using high-resolution magnetic resonance imaging. Br J Surg. 2003 Mar;90(3):355-64.

4. Brown G, Richards CJ, Bourne MW, Newcombe RG, Radcliffe AG, Dallimore NS, Williams GT. Morphologic predictors of lymph node status in rectal cancer with use of high-spatial-resolution MR imaging with histopathologic comparison. Radiology. 2003 May;227(2):371-7.

5. Engelen SM, Beets-Tan RG, Lahaye MJ, Kessels AG, Beets GL. Location of involved mesorectal and extramesorectal lymph nodes in patients withprimary rectal cancer: preoperative assessment with MR imaging. Eur J Surg Oncol. 2008 Jul;34(7):776-81.

6. Lahaye MJ, Beets GL, Engelen SM, Kessels AG, de Bru√Øne AP, Kwee HW, van Engelshoven JM, van de Velde CJ, Beets-Tan RG. Locally advanced rectal cancer: MR imaging for restaging after neoadjuvant radiation therapy with concomitant chemotherapy. Part II. What are the criteria to predict involved lymph nodes? Radiology. 2009 Jul;252(1):81-91.

7. MERCURY Study Group. Relevance of magnetic resonance imaging-detected pelvic sidewall lymph node involvement in rectal cancer. Br J Surg. 2011 Sep 16.

8. Eisenhauer EA, Therasse P, Bogaerts J, Schwartz LH, Sargent D, Ford R, Dancey J, Arbuck S, Gwyther S, Mooney M, Rubinstein L, Shankar L, Dodd L, Kaplan R, Lacombe D, Verweij J New response evaluation criteria in solid tumours: revised RECIST guideline (version 1.1). Eur J Cancer. 2009 Jan;45(2):228-47

9. Patel UB, Brown G, Rutten H, West N, Sebag-Montefiore D, Glynne-Jones R, Rullier E, Peeters M, Van Cutsem E, Ricci S, Van de Velde C, Kjell P, Quirke P. Comparison of magnetic resonance imaging and histopathological response to chemoradiotherapy in locally advanced rectal cancer. Ann Surg Oncol. 2012 Sep;19(9):2842-52. doi: 10.1245/s10434-012-2309-3. Epub 2012 Apr 24.

10. Lambregts DM, Lahaye MJ, Heijnen LA, Martens MH, Maas M, Beets GL, Beets-Tan RG. MRI and diffusion-weighted MRI to diagnose a local tumour regrowth during long-term follow-up of rectal cancer patients treated with organ preservation after chemoradiotherapy. Eur Radiol. 2015 Oct 30.

11. Martens MH, van Heeswijk MM, van den Broek JJ, Rao SX, Vandecaveye V, Vliegen RA, Schreurs WH, Beets GL, Lambregts DM, Beets-Tan RG. Prospective, Multicenter Validation Study of Magnetic Resonance Volumetry for Response Assessment After Preoperative Chemoradiation in Rectal Cancer: Can the Results in the Literature be Reproduced? Int J Radiat Oncol Biol Phys. 2015 Dec 1;93(5):1005-14.

12. Beets-Tan RGH, Lambregts DMJ, Maas M, Bipat S, Barbaro B, Curvo-Semedo L, Fenlon HM, Gollub MJ, Gourtsoyianni S, Halligan S, Hoeffel C, Kim SH, Laghi A, Maier A, Rafaelsen SR, Stoker J, Taylor SA, Torkzad MR, Blomqvist L. Magnetic resonance imaging for clinical management of rectal cancer: Updated recommendations from the 2016 European Society of Gastrointestinal and Abdominal Radiology (ESGAR) consensus meeting.Eur Radiol. 2018 Apr;28(4):1465-1475

13. Jang JK, Lee JL, Park SH, Park HJ, Park IJ, Kim JH, Choi SH, Kim J, Yu CS, Kim. Magnetic resonance tumour regression grade and pathological correlates in patients with rectal cancer. JC. Br J Surg. 2018 Nov;105(12):1671-1679.

14. Lee MA, Cho SH, Seo AN, Kim HJ, Shin KM, Kim SH, Choi GS.Modified 3-Point MRI-Based Tumor Regression Grade Incorporating DWI for Locally Advanced Rectal Cancer. AJR Am J Roentgenol. 2017 Dec;209(6):1247-1255. doi: 10.2214/AJR.16.17242. Epub 2017 Oct 5.

15. Sclafani F, Brown G, Cunningham D, Wotherspoon A, Mendes LST, Balyasnikova S, Evans J, Peckitt C, Begum R, Tait D, Tabernero J, Glimelius B, Roselló S, Thomas J, Oates J, Chau I. Comparison between MRI and pathology in the assessment of tumour regression grade in rectal cancer. Br J Cancer. 2017 Nov 7;117(10):1478-1485. doi: 10.1038/bjc.2017.320. Epub 2017 Sep 21.
