# Supplementary material for: Preoperative short-course radiation therapy with PROtons compared to photons in high-risk RECTal cancer (PRORECT): Initial dosimetric experience
Source: Clin Transl Radiat Oncol. 2022 Dec 17;39:100562. doi: 10.1016/j.ctro.2022.100562 (PMC9792362; doi:10.1016/j.ctro.2022.100562)
Supplement: Supplementary data 3 [file mmc3.pdf]

Subsites 1-4 are mandatory, additional subsites added depending on tumor features and patient features<br>Modified from Valentini et al, PMID 27528121 and Ng et al, PMID 22401917<br>Isotropic CTVN is delineated on MR<br>OAR: S1-S2, bladder, bowel bag, femoral heads. **OARs are delineated on MR, bowel bags delineated on CT**. PTV is added by a physicist			PRORECT
**Subsite**	**Limits CTVN**<br>**7-8 mm marginal to vessels minus bone/muscle if not stated otherwise**	**Limits ITVN**	**Recommendations:**
**1** Primary tumor	**15 mm marginal from GTVT to CTVT or zCTVT**		Mandatory
**2** Presakral subsite, pelvic part	**Cranial limit**: bifurcation a. iliaca ext/int or promotory<br>**Caudal limit**: caudal limit mesorectum		Mandatory<br>Consider lowering cranial limit to S2-S3 if ≤T3,N0,MRF- and non-high T.*<br>Always at least 20 mm from cranial limit GTVT to cranial limit CTVN.
**3** Mesorectum	**Cranial limit**: rectosigmoidal junction<br>**Caudal limit:** insertion of the m. levator ani into the external sphincter (disappearing of the mesorectal fat)<br>**Posterior limit**: presacral subsite until beginning of fossa ischiorectalis<br>Mesorectal fascia in other directions	Isotropic CTV: Anteriorly add up to 10 mm to account for bladder filling variations. Consider the same if T4 is adjacent to uterus/vesicles. Posteriorly, in low T, account for pitching of the pelvis and add 5 mm.	Mandatory
**4** Lateral lymphatic nodes, a. iliaca interna subsite (situated laterally of mesorectum and presacral subsite)	**Cranial limit**: bifurkation a. iliaca externa/interna<br>**Caudal limit**: insertion of the m. levator ani into the external sphincter (disappearing of the mesorectal fat)<br>**Anterior limit**: 7-8 mm around vessels in the upper pelvis. Mid pelvis: up to external vessels. Lower pelvis: approximately middle of m obturatorius<br>**Lateral limit**: m iliopsoas, m piriformis, m obturatorius int and bone.		Mandatory<br>Consider lowering cranial limit to S2-S3 if ≤T3, N0, MRF- and non-high T.*<br>Always at least 20 mm from cranial limit GTVT to cranial limit CTVN.
**5** Lateralt lymphatic nodes, obturator subsite (small volume)	**Cranial limit:** 5 mm above canalis obturatorius<br>**Caudal limit:** canalis obturatorius<br>**Anterior limit**: widest part of femoral head<br>**Medial limit**: bladder		Include in case of:<br>Positive nodes in the a iliaca interna subsite,<br>T4,<br>N2<br>T below the peritoneal fold

**6** Lateral lympatic nodes, a.iliaca externa subsite	**Cranial limit**: bifurcation a iliaca externa/interna<br>**Caudal limit**: between roof of acetabulum and ramus pubis superior<br>**Anterior limit**: 15 mm anterolaterally along the m iliopsoas		Infiltration of anterior organs, i.e prostate, vesicles, uterus, bladder, vagina or positive nodes in the obturatory- or ingual subsites
**7** Sphincter	**Cranial limit**: Anorectal juncion–>entire sphincter included		Infiltration of sphincter/analcanal
**8** Fossa ischiorectalis (FIR)	Triangel of adipose tissue limited by levator ani, m obturatorius and m gluteus, posteriorly until m gluteus ”turns”.<br>**Caudal limit**: anal verge (sphincter included) in a plane of tuberositas ishii		Infiltration of external sphincter, levators or fossa ischiorectalis
**9** Inguinal lymphatic nodes. Pars profunda et superficialis	**Cranial limit**: where the external vessels leave the pelvis<br>**Caudal limit**: tuberositas ishii<br>**Anteriomedial limit**: at least 20 mm, include visible nodes/lymphocele<br>**Posterior limit**: adjacent muscles		Include in case of:<br>Positive inguinal nodes, infiltration below linea dentata, massiv infiltration of the external sphincter or infiltration of lower vagina
**10** Iliaca communis subsite	**Cranial limit**: aortal bifurcation and/or 5 mm above positive nodes<br>**Caudal limit**: bifurcation a iliaca externa/interna		To be included if decided by the tumor board
**Explanation to subsite 2 och 4**			*In accordance to Valentini et al.<br>Consider lowered cranial limit if ≤T3 and non-high T (10-15 cm from anal verge) :<br><br>MFR- with only mesorectala positive nodes: S1/S2 (Syk et al PMID 18495376)<br>MRF- with N0: S2/S3 (Nijkamp et al PMID 20646849)<br><br>ALWAYS at least 20 mm from cranial limit GTVT to cranial limit CTVN.
