# Supplementary material for: Preoperative short-course radiation therapy with PROtons compared to photons in high-risk RECTal cancer (PRORECT): Initial dosimetric experience
Source: Clin Transl Radiat Oncol. 2022 Dec 17;39:100562. doi: 10.1016/j.ctro.2022.100562 (PMC9792362; doi:10.1016/j.ctro.2022.100562)
Supplement: Supplementary data 4 [file mmc4.pdf]

Preoperative Short-Course Radiation Therapy With PROtons Compared to Photons In High-Risk RECTal Cancer (PRORECT): A Prospective Randomized Swedish Phase II Trial

Synopsis of the protocol

**Protocol title**	Preoperative Short-Course Radiation Therapy With Protons Compared to Photons in High-Risk Rectal Cancer (PRORECT): A Prospective Randomized Swedish Phase II Study (NCT04525989)
**Protocol Phase**	Phase II
**Indication**	Primary rectal cancer with high risk of failing locally and/or systemically
**Background**	In patients with a newly diagnosed locally advanced rectal cancer (LARC) at high risk of local or systemic relapse, long-course preoperative radiotherapy with concomitant chemotherapy followed by surgery after 6-8 weeks has been standard therapy.<br><br>Total neoadjuvant therapy (TNT) is a novel approach for LARC, which delivers both systemic chemotherapy and neoadjuvant (chemo)radiotherapy prior to surgery. There is a growing interest in treating LARC with TNT after recently published randomized phase III trials RAPIDO and STELLAR. Short-course radiotherapy (SCRT) used in these studies was pioneered in Sweden and has since gained increased acceptance in treatment of LARC. Recent ESMO and NCCN guidelines list SCRT followed by systemic chemotherapy as an option for preoperative treatment in LARC.<br><br>Neoadjuvant radiotherapy with protons has advantages over photon therapy in dosimetric treatment planning studies. When compared to photon plans, radiotherapy with proton reduced small bowel and femur dose in clinical re-irradiation studies suggesting the presence of clinical benefit in terms of reduced toxicity. There are also some suggestions that proton dosimetry may be particularly better for larger tumors.<br><br>The aim of this study is to study whether **proton therapy** in locally advanced rectal cancer can offer meaningful reductions in acute

	gastrointestinal toxicity compared to **standard treatment with photons** which may improve patient's tolerability of neoadjuvant chemotherapy.<br>There are currently no published clinical reports evaluating the use of proton therapy in the upfront treatment of locally advanced rectal cancer. There are further no published randomized trials comparing radiotherapy with photon vs proton in locally advanced rectal cancer.
**Endpoints**	**Primary endpoint:**<br>• The incidence of acute preoperative grade 2-5 gastrointestinal toxicity according to CTCAE v5.0 (Appendix 3) associated with proton vs. photon radiotherapy<br>(Time Frame: from start of radiotherapy to planned start of the third (3) CAPOX cycle (week 9-10 of the trial).<br><br>**Secondary endpoints:**<br>• To estimate the incidence of all toxicity (hematologic and non-hematologic) associated with protocol treatment in the preoperative period, the postoperative period, and overall.<br>• To determine differences in patient reported outcomes (PRO) between treatment arms in the preoperative period, the postoperative period, and overall<br>• To determine differences between treatment arms in proportion of patients being able to undergo full dose neoadjuvant chemotherapy i.e. at least 4 cycles of CAPOX or 6 cycles of FOLFOX<br>• To radiologically assess and compare tumour regression grading (mrTRG) between treatment arms<br>• Health economic comparison between proton and photon treatment. Cost effectiveness analysis measured by QALY<br>**Safety endpoints:**<br>• Disease free survival after proton vs. photon treatment<br>• Overall survival after proton vs. photon treatment<br>• Quality of life after proton vs. photon treatment (QLQ-C30)<br>• Difference in late postoperative complications between study arms according to LARS score (1)<br>• Proportion of patients who reach a clinical complete remission (cCR) after chemoradiotherapy, enter a watch-and-wait period and remain free of regrowth at least one year<br>• To determine differences in acute neurogenic pain during proton vs. photon treatment (Time Frame: from start of radiotherapy to end of radiotherapy)<br>**Exploratory endpoints:**<br>• To determine differences between treatment arms in concentrations of CD8+ and FOXP3 + tumor-infiltrating T cells after given radiotherapy

**Study design**	Patients will be treated with the short-course 5 x 5 Gy radiation scheme (photons or protons) followed by planned at least four cycles of combination chemotherapy (capecitabine and oxaliplatin) and TME surgery. The planned number of chemotherapy courses may vary from four to six. An alternative is to give a comparable number of cycles giving FOLFOX.
**Total number of centres**	All seven Swedish University Hospitals participating in the Skandion clinical network
**Selection criteria**	Patients with a primary rectal cancer without detectable distant metastasis who after locoregional therapy only, meaning preoperative radio(chemo) therapy plus surgery have at least a 40% risk of having a CRM positive resection or a recurrence, local or distant, within three years.
**Main criteria for inclusion**	**Primary tumour characteristics (identical to those of the RAPIDO and LARCT-US trials):**<br>• Histological proof of newly diagnosed primary adenocarcinoma of the rectum.<br>• Locally advanced tumour fulfilling at least one of the following criteria on pelvic MRI indicating high risk of failing locally and/or systemically: T4b, i.e. infiltration of an adjacent organ or structure like the prostate, urinary bladder, uterus, sacrum, pelvic floor or side-wall (according to TNM version 8), cT4a, i.e. peritoneal involvement, extramural vascular invasion (EMVI+), N2-status regarded as metastatic according to ESGAR consensus criteria (2) (see Radiology Appendix), positive MRF, i.e. tumour or lymph node ≤ 1 mm from the mesorectal fascia, enlarged lateral nodes (lat LN+ according to ESGAR consensus criteria (2), see Radiology Appendix)<br><br>**General:**<br>• Staging done within 6 weeks before start of radiotherapy.<br>• No contraindications to chemotherapy with CAPOX or FOLFOX, including adequate blood counts:<br>- white blood count ≥4.0 x 10^9/L<br>- platelet count ≥100 x 10^9/L<br>- clinically acceptable haemoglobin levels<br>- creatinine levels indicating renal clearance of ≥50 ml/min<br>- bilirubin <35 µmol/l.<br>• ECOG performance score ≤ 1.<br>• Patient is considered to be mentally and physically fit for chemotherapy as judged by the oncologist.<br>• Age ≥ 18 years<br>• Written informed consent.<br>• Adequate potential for follow-up.

**Exclusion criteria**	• See detailed description in the protocol
**Main parameters of efficacy**	**Primary:** Acute preoperative gastrointestinal toxicity at week 9-10 of the trial
	**Secondary**: Acute and late side effects; Adverse events and side effects graded according to CTCAE v5.0 (Appendix 3 and Appendix 4).<br>Proportion of patients starting chemotherapy within 14 weeks after the first radiation fraction and able to undergo the full treatment schedule (at least 4 CAPOX/6 FOLFOX)
**Screening**	Baseline screening includes:<br>• CT (or MRI) of the abdomen and liver<br>• MRI of the pelvis<br>• CT of the thorax<br>• Routine blood tests
**Treatment**	**Standard Treatment (Arm A):**<br>Week 1: 5 x 5 Gy External Radiation Therapy with Photons<br>Week 3-14 4 courses of CAPOX (Capecitabine b.i.d.1000 mg/m^2 day 1-14 every 3 weeks, Oxaliplatin 130 mg/m^2 day 1 every 3 weeks)<br>Week 17-20: Surgery (TME)<br><br>**Experimental Treatment (Arm B):**<br>Week 1: 5 x 5 Gy (RBE=1.1) External Radiation Therapy with Protons<br>Week 3-14: 4 courses of CAPOX (Capecitabine b.i.d.1000 mg/m^2 day 1-14 every 3 weeks, Oxaliplatin 130 mg/m^2 day 1 every 3 weeks)<br>Week 17-20: Surgery (TME)
**Statistical considerations**	Two independent study groups<br>Primary endpoint: dichotomous<br>Power: 80%, 5% type 1 error<br>Rate of anticipated grade 2-5 GI-toxicity in standard group: 30%<br>Rate of anticipated all-grade GI-toxicity in experimental group: 15%<br>Dropout rate: 5 %<br>Sample size: 127 patients in each group<br>Total number of patients: 254<br><br>Incidence of adverse events as assessed by Common Terminology Criteria for Adverse Events version 5 [Time Frame: From baseline up to 5 years]<br>A Chi-square test will be used to compare the number of patients with at least 1 grade 2 or higher adverse events between the treatment arms.

	Fatigue as measured by MFI.<br>Anxiety and depression measured by HAD (Forms Attached)<br>[Time Frame: From baseline to week 3, the assessment at the start of CAPOX or 1 month, months 3, 6, 9, 12, 24, 36, 60)<br>Change in fatigue will be compared between treatment arms using a t-test. If the data do not satisfy the normality assumption, a Wilcoxon test may be used instead. P-values <=0.05 will be assumed to indicate statistical significance.<br><br>DFS, PFS and overall survival will be analyzed using the Kaplan-Meier approach, comparing the arms by log-rank test.<br><br>In a subsequent step, Cox regression will be used to address the relative risk of clinical factors on overall survival<br><br>**Efficacy**: Stratified Cox regression analysis<br>**Safety**: Mann-Whitney U-test
**Planned sample size**	The number of patients to be included has not been fixed, but it is estimated to be at least 254
**Analysis plan**	The primary endpoint will be analysed when the last patient has completed treatment including the surgery. For patients who are not operated because of a cCR, minimal follow-up for safety should be 12 months.
**Duration of the study**	Inclusion during two to three years, five years follow-up after inclusion of the last patient
