# Supplementary material for: Preoperative short-course radiation therapy with PROtons compared to photons in high-risk RECTal cancer (PRORECT): Initial dosimetric experience
Source: Clin Transl Radiat Oncol. 2022 Dec 17;39:100562. doi: 10.1016/j.ctro.2022.100562 (PMC9792362; doi:10.1016/j.ctro.2022.100562)
Supplement: Supplementary data 5 [file mmc5.pdf]

Radiotherapy Appendix

Patient pre-treatment preparation

All preparation with fixation will be done at one of the university hospitals participating in the Skandion network. A custom lower limb immobilization device for supine patients is required to minimize setup uncertainty.

To minimize bladder volume variability, the patients will be subjected to following procedure: after voiding, the patients will be asked to drink 300 cc liquid. Pre-treatment imaging will be performed 45 minutes afterwards. The same routine will be repeated before each treatment fraction.

Pre-treatment imaging

Dose-planning CT and MR will be done at one of the university hospitals participating in the Skandion network. Maximum acceptable rectum diameter on planning imaging is 5 cm. For proton and photon treatment planning, dose calculations must be performed on a CT study without contrast. The CT scanning protocol must be validated for protons and photons.

Target Volumes

Target definition and dose-planning will be done at one of the university hospitals participating in the Skandion network. Structure delineation will be performed on a registered CT/MR image.

The definition of volumes will be done in accordance with the ICRU Report #83: Prescribing, Recording and Reporting Photon Beam Therapy (1), and ICRU #78 (2)

The Gross Tumor Volume (GTV) is defined as all known gross disease as determined from a combination of physical exam, colonoscopy, CT, MRI and PET-CT if performed.

The Clinical Target Volume (CTV) is defined as the GTV plus areas considered at significant risk of harboring microscopic disease.

The Planning Target Volume (PTV) will provide a margin around the CTV to compensate for the inter- and intra-fraction uncertainty consequent to daily setup uncertainty and to potential internal organ motion. By definition, the PTV will consist of a symmetrical 6 mm expansion around the CTV. In the event that PTVs extend outside of the skin surface, the clinician should manually trim the PTV contours to be 3-5 mm inside the outer skin (unless there is direct skin involvement).

The following are guidelines for generating CTV and a unified PTV.
Rectal GTV (+15 mm radially and 20 mm cranio-caudally) = CTV

Nodal GTV + 10 mm symmetrical expansion = CTV
Uninvolved iliac vessels + 7-8 mm= CTV

For details regarding target volume definition see "Target volumes Appendix"

During treatment optimization help structures with customized margins to the CTV can be created in order to include range uncertainties and to ensure homogeneous and robust dose delivery to the CTV.

Organs at risk (OARs)

OARs will be delineated in accordance with the national Swedish guidelines (STRÅNG-project) (3, 4). Bladder, bowel bag, femoral heads, sacral nerves, pelvic bones will be defined. Pelvic bone will be auto contoured for the full extent of the PTV. For protons, artifacts in the tissue as well as clips, markers, etc. must also be contoured and replaced with appropriate Hounsfield unit (HU).

Planning Constraints

**Structure**	**Priority**	**Dose constraint (Gy, RBE)**	**Description**
Bowel bag (5, 6)	2	$V_{18Gy} \leq 450cc$	The volume receiving 18 Gy (RBE) should be equal or less than 450 cc
Femoral heads	2	$D_{mean} < 25$ Gy	The mean dose should be less than 25 Gy (RBE)
Sacrum (spinal canal at the level of S1-S2). Definition of lumbosacral plexus (7) is optional	2	$V_{25Gy} < 60\%$	The volume receiving 25Gy (RBE) should be less than 60%
Pelvic Bones	3	As low as reasonably achievable. Aviod hotspots	Total bone for the full extent of the PTV
Bladder	3	As low as reasonably achievable. Avoid hotspots	

Table 1. *Dose-volume constraints. RBE value/model 1.10 is used.*

Absorbed dose prescription

All patients will receive 5 fractions of 5 Gy (RBE) 1 fraction per day, five days per week up to a total dose of 25 Gy (RBE). Treatment starts preferably on Monday. Overall treatment time should be maximum eight days.

Doses that have been reduced due to toxicity must never be re-escalated.

The prescribed absorbed dose should be specified to a dose reference point/volume.

Standard treatment with photons. Arm A:

Preoperative radiotherapy will be delivered on a linear accelerator. The use of a belly board is allowed. Intensity modulated radiation therapy (IMRT) is mandatory for all patients for the initial pelvic field encompassing the gross tumor and at-risk lymph nodes in the pelvis.

The dose distribution should be calculated based on CT scans or CT-scans registered with MRI and specified according to the ICRU 83 guidelines.

PTV planning dose-volume constraint	Priority	Description
V93% ≥ 98%	1	≥ 98% of the PTV receives ≥ 93% of the prescribed dose
V105% ≤ 10%	1	≤ 10% of the PTV receives ≥ 105% of the prescribed dose
V110% ≤ 5%	1	≤ 5% of the PTV receives ≥ 110% of the prescribed dose
V115% < 0%	1	None of the PTV is to receive ≥ 115% of the prescribed dose

Table 2. *PTV planning dose-volume constraints*

Experimental treatment with protons. Arm B:

RT plans will generally consist of either (1) a single PA (posterior-anterior) field to encompass the primary tumor, mesorectum, and internal iliac lymph nodes, with a single AP (anterior-posterior) field to treat the external iliac lymph nodes or (2) posterior oblique RT fields. Single-field optimization will be used with intensity modulated gradients for regions of overlap, or a (3) 3-field (posterior/anterior, right lateral, and left lateral) approach with a 2-to-1 field weighting by dose contributed to the target volume. To account for air within the rectum when designing the proton plan, the Hounsfield units need to be overridden for the circumferential air-filled portion of the rectum.

Proton treatments plans will be defined by the same principles as for photons and used for prescribing and reporting dose according to the recommendations of ICRU (8). During treatment optimization help structures with customized margins to the CTV can be created in order to include range uncertainties and to ensure homogeneous and robust dose delivery to the CTV.

For proton therapy, the dose prescriptions should be in both physical dose and RBE-weighted absorbed doses. The RBE value of 1.1 should be used for protons and the prescribed dose is the corresponding dose for photons (Gy(RBE)).

Treatment planning and delivery

Treatment technique
The radiation treatment can be given with photons (IMRT/VMAT) or protons.

Dose computation and optimization

Dose calculation

o Reference dosimetry is carried out according to the IAEA report TRS 398 (2000) (9)

o The absorbed dose in the patient geometry shall be calculated by using validated algorithms.

o The calculation grid shall be at a maximum of 3 mm.

In order to facilitate the optimization procedure, help structures and volumes may be defined.

For proton treatments, see treatment planning and optimization guidelines in the Skandion Clinic treatment planning instruction manual.

Treatment plan evaluation

A robustness test should be performed for each proton treatment in 14 test cases according to the recommendations in the Skandion clinic treatment planning instruction manual. Tolerance levels that should be fulfilled in the robustness test for treatments described in table 3.

	**Tolerance levels**
CTV: all test cases	D90% ≥ 95%
CTV: for at least 12 of 14 robustness test cases	D98% ≥ 95%
GTV: all test cases	D95% ≥ 100%
GTV: for at least 12 of 14 robustness test cases	D98% ≥ 100%
DVH parameters of OARs used for definition of objectives and constraints (table 1)	According to clinical judgement

Table 3. *Tolerance levels that should be fulfilled in the robustness test. RBE value/model 1.10 is used.*

Image-guided treatment delivery

The usage of IGRT is essential since the total number of fractions is limited to five. For both protons and photons, the position of the patient shall be verified daily based on CBCT.

Additionally, the optical surface scanning system Catalyst ™ (C-RAD, Uppsala, Sweden) can be used as a complement for positioning and monitoring the patient intrafractionally.

CBCT is used for daily positioning and corrections are made in 6DoF daily, accordingly. Auto match in the system is applied followed by manual adjustment. For improved definition, localization and monitoring of target and OAR position, CBCT images of each fraction are evaluated daily, both online and offline by a physician and a physicist. Anatomy including surface/skin, bone, gas, urinary bladder as well as the CTV is evaluated. If adjustments outside the approved margins are necessary, a CBCT scan or kV-images are repeated after adjusting the position for verification.

For proton therapy; translational correction ≥ 8mm and rotational corrections ≥ 2°.

If large anatomical changes can be seen on the CBCT and replanning is deemed necessary, a CT scan is made in direct connection to the treatment and the CT-scan is used for a new or adjusted treatment-plan.

In-vivo dosimetry

The absorbed dose to the patient shall be estimated based on in vivo measurements according to clinical routine procedures.

If in-vivo dosimetry is not applicable, pre-treatment patient specific quality assurance (QA) must be performed.

Quality assurance

The aim of the RT-QA program is to ensure the consistency of radiotherapy treatment delivery across all participating centers as well as the verification of adherence to the protocol guidelines described above.

Radiotherapy-related data will be collected, transferred and reported to QA-centre by each participating site. The data transfer is handled through the Sharefile service provided by the Skandionkliniken https://skandionbasa.sharefile.eu/

Each participating center has the access to Sharefile folder “PRORECT NNN”, where NNN is the name of the centere. For each patient a subdirectory to “PRORECT XX” should be created and named “XX-YYY”, where XX-YYY is the patient identification number (XX defines participating centre and YYY is a running patient number).

Following data from the TPS are

1. Exported in DICOM-format and saved under the directory “XX-YYY”

- CT-images
- Structures
- Treatment plan
- Total dose distribution (in Gy) in DICOM-format
- Co-registered MR of PET-images, when applicable

2. Saved as a document (Word, Excel, Text of .pdf) stating the date of the first and last treatment fractions. The file should be named "XX-YYYY" and saved in the directory "XX-YYY"

The RT QA program is divided in two moments:

1. **Pre-study RT-QA (before inclusion of patients)**

Benchmark cases
All participating centers are required to delineate the target volume and the organs at risk on CT images from two test cases. The delineation should be done according to the above instructions.

Additionally, all centers are required to submit a radiotherapy plan for each technique on two further test cases with predefined targets and organs at risk. The radiotherapy plans should respect the dose constraints described above.

All the benchmark cases will be evaluated by the RT-QA coordinating center before the participating centers can proceed with the patient inclusion.

Dosimetry audit
In order to check the absolute dosimetry of the accelerators that will be used to deliver the treatment, it is recommended that all participating centers should take part in an external dosimetry audit alternatively to provide a reasonable recent documentation about it.

2. **During study RT-QA**

In order to maintain the compliance to the RT protocol throughout the duration of the study, the RT-QA coordinating centre will keep performing the evaluation according to the following steps:

Pre-treatment review:

This stage is composed of two levels:

- Level 1:
  Within this level, evaluation of the delineation (target and organs at risk) and of the treatment plan will be performed.
  The first radiotherapy plan from each treating centre, for photon respectively proton treatment, will be reviewed. All centres are required to send RT data at least one week, counted as five working days, before the start of the treatment. Feed-back to the treating centre will be provided within three working days from reception. If the treatment plans are within the centre can start the treatment as planned and progress to level 2.
  If major violations are identified a re-planning will be requested before the start of the treatment. The modified plan must be submitted for review, but approval will not be

required prior to the start of the treatment unless otherwise specified by the RT-QA committee. For these centres, one more patient will be reviewed according to the above-described procedure.
Once an acceptable quality level is achieved, centres may progress to level 2.

- Level 2:
  Within this level evaluation of the treatment plans will be performed and RT data is to be sent the first day of the treatment at the latest.
  All centres will keep being audited with one in every five patients randomly selected by the RT-QA committee for review. However, preapproval from the RT-QA coordinating centre will not be required to begin the treatment.
  If a continued level of acceptable quality is maintained, the rate of sampling may be decreased at the discretion of the RT-QA committee.

Continuous monitoring of RT treatments:
For the rest of the patients included in the study, a continuous monitoring of RT data will be performed and therefore it is required that all RT data should be sent the first day of the treatment at the latest. If major deviations are found under the period of the study the RT-QA coordinating center will provide feedback. This will, however, not affect ongoing treatments.

All centers are required to submit the final RT data of all involved patients within two weeks after completion of the RT treatment.

Documentation

Detailed information on how to prepare, process and send the RT data for all involved patients will be provided separately. Likewise, instructions on feedback communication from the RT-QA coordinating system will be given separately.

References:

1. Measurements TICoRUa. Prescribing, Recording and Reporting Photon Beam Therapy. ICRU report 50.
2. Measurements ICoRUa. Prescribing, Recording and Reporting Proton-Beam Therapy. ICRU Report 78. 2007.
3. Olsson C, Nyholm T, Wieslander E, Onjukka E, Gunnlaugsson A, Reizenstein J, et al. Initial experience with introducing national guidelines for CT- and MRI-based delineation of organs at risk in radiotherapy. Physics and Imaging in Radiation Oncology. 2019;11:88-91.
4. Nyholm T, Olsson C, Agrup M, Bjork P, Bjork-Eriksson T, Gagliardi G, et al. A national approach for automated collection of standardized and population-based radiation therapy data in Sweden. Radiother Oncol. 2016;119(2):344-50.
5. Devisetty K, Mell LK, Salama JK, Schomas DA, Miller RC, Jani AB, et al. A multi-institutional acute gastrointestinal toxicity analysis of anal cancer patients treated with concurrent intensity-modulated radiation therapy (IMRT) and chemotherapy. Radiother Oncol. 2009;93(2):298-301.
6. Gay HA, Barthold HJ, O'Meara E, Bosch WR, El Naqa I, Al-Lozi R, et al. Pelvic normal tissue contouring guidelines for radiation therapy: a Radiation Therapy Oncology Group consensus panel atlas. Int J Radiat Oncol Biol Phys. 2012;83(3):e353-62.
7. Tunio M, Al Asiri M, Bayoumi Y, Abdullah OBA, AlHameed M, Gabriela SL, et al. Lumbosacral plexus delineation, dose distribution, and its correlation with radiation-induced lumbosacral plexopathy in cervical cancer patients. Onco Targets Ther. 2015;8:21-7.
8. ICRU. Prescribing, Recording, and Reporting Proton-Beam Therapy (ICRU Report 78).
9. Agency IAE. Absorbed Dose Determination in External Beam Radiotherapy. An International Code of Practice for Dosimetry Based on Standards of Absorbed Dose to Water. IAEA TRS 398. 2000.
